# The genome sequence of the Diamond-back Marble, *Eudemis profundana* (Denis & Schiffermüller, 1775)

**DOI:** 10.12688/wellcomeopenres.19370.1

**Published:** 2023-04-25

**Authors:** Douglas Boyes, James Hammond, University of Oxford and Wytham Woods Genome Acquisition Lab, Darwin Tree of Life Barcoding collective, Wellcome Sanger Institute Tree of Life programme, Wellcome Sanger Institute Scientific Operations: DNA Pipelines collective, Tree of Life Core Informatics collective, Darwin Tree of Life Consortium

**Affiliations:** 1 UK Centre for Ecology & Hydrology, Wallingford, England, UK; 2 University of Oxford, Oxford, England, UK

**Keywords:** Eudemis profundana, Diamond-back Marble, genome sequence, chromosomal, Lepidoptera

## Abstract

We present a genome assembly from an individual male *Eudemis profundana* (the Diamond-back Marble; Arthropoda; Insecta; Lepidoptera; Tortricidae). The genome sequence is 691.3 megabases in span. Most of the assembly is scaffolded into 28 chromosomal pseudomolecules, including the Z sex chromosome. The mitochondrial genome has also been assembled and is 16.5 kilobases in length.

## Species taxonomy

Eukaryota; Metazoa; Ecdysozoa; Arthropoda; Hexapoda; Insecta; Pterygota; Neoptera; Endopterygota; Lepidoptera; Glossata; Ditrysia; Tortricoidea; Tortricidae; Olethreutinae; Olethreutini; *Eudemis*; *Eudemis profundana* (Denis & Schiffermüller, 1775) (NCBI:txid1100989).

## Background

*Eudemis profundana* (Denis & Schiffermüller, 1775) is a moth of the Tortricidae family. A large and robust species for its family, *profundana* shows a wide range of variation in colouration of the forewing, particularly in the strength of its white markings ( Bradley *et al.*, 1979). The species frequents oak ( *Quercus*) woodland, and adult moths fly high up amongst foliage before sunset, resting on branches and trunks of oak trees by day, The larva feeds on oak leaves, rolling the leaf around the leaf’s midrib, between May and June. Pupation occurs either in the larval habitation or amongst leaf litter on the ground. Adults can be found between June and September ( Bradley *et al.*, 1979; Elliott *et al.*, 2018).

This species is widespread across the British Isles, reaching southern Scotland, and being common in the oak woodlands of southern Ireland ( Bradley *et al.*, 1979; Elliott *et al.*, 2018). Globally the moth is found across Eurasia eastwards to at least the Caucasus ( Maharramova & Ayberk, 2017). The species is also reported from Hokkaido, and the Korean peninsula, being known to feed on *Prunus ssiori* in Hokkaido, alongside *Quercus* ( Bae & Sakamaki, 1995). However, specimens formerly identified as *profundana* in the Korean peninsula are now regarded as *Eudemis lucina* or *E. brevisetosa* ( Sohn *et al.*, 2015), so these records may not necessarily refer to *profundana*.

The genome of *Eudemis profundana* was sequenced as part of the Darwin Tree of Life Project, a collaborative effort to sequence the named eukaryotic species in the Atlantic Archipelago of Britain and Ireland. Here we present a chromosomally complete genome sequence for *Eudemis profundana*, based on one male specimen from Wytham Woods, Oxfordshire, UK.

## Genome sequence report

**Figure 1.**
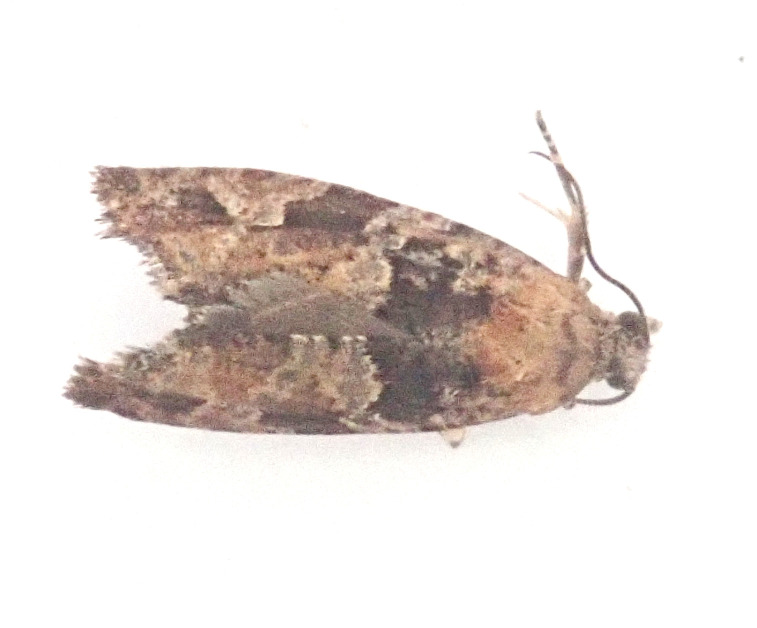
Photograph of the *Eudemis profundana* (ilEudProf1) specimen used for genome sequencing.

The genome was sequenced from one male *Eudemis profundana* ( Figure 1) collected from Wytham Woods, Oxfordshire, UK (latitude 51.77, longitude –1.31). A total of 37-fold coverage in Pacific Biosciences single-molecule HiFi long reads was generated. Primary assembly contigs were scaffolded with chromosome conformation Hi-C data. Manual assembly curation corrected 56 missing joins or mis-joins and removed 17 haplotypic duplications, reducing the assembly length by 0.59% and the scaffold number by 56%.

**Figure 2.**
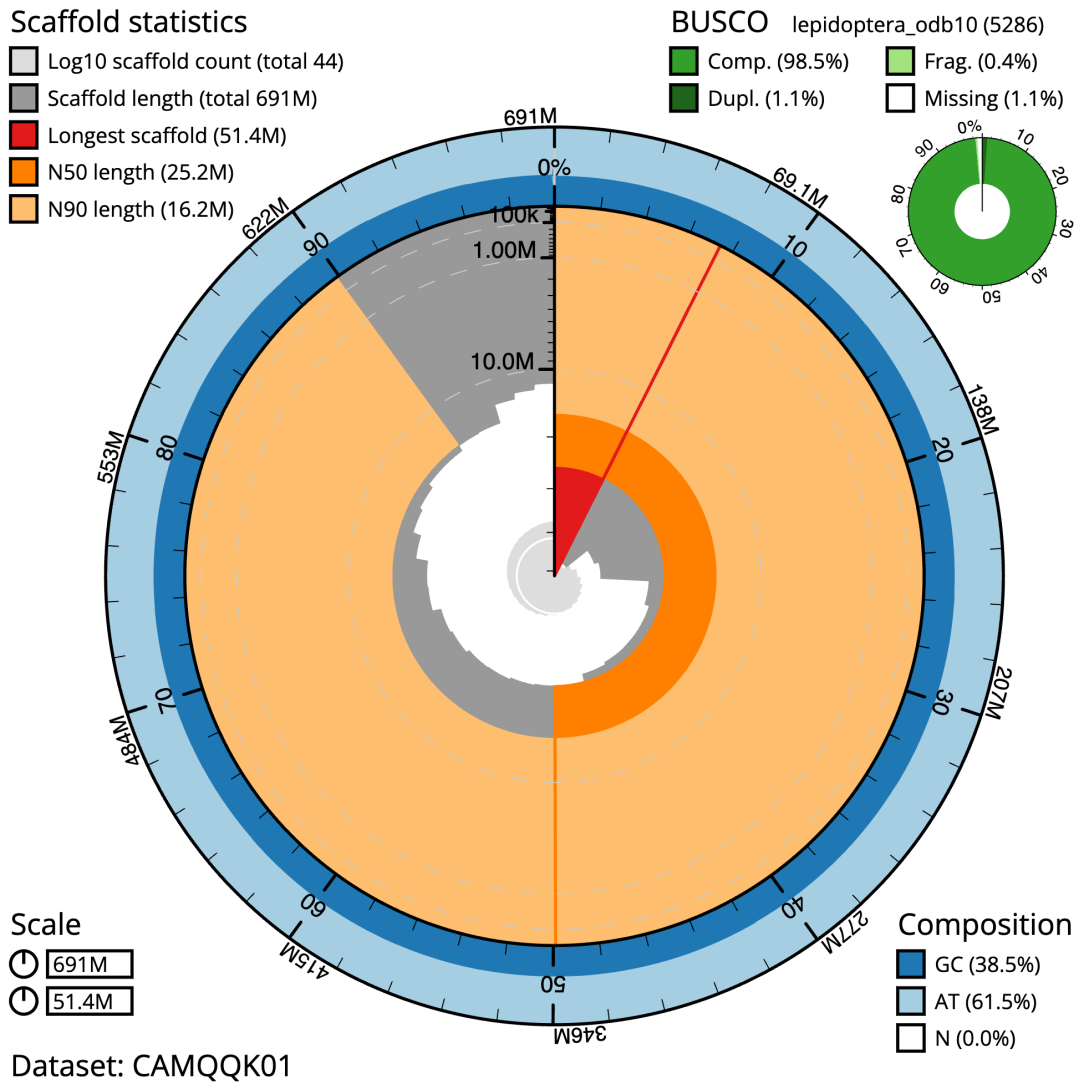
Genome assembly of *Eudemis profundana*, ilEudProf1.1: metrics. The BlobToolKit Snailplot shows N50 metrics and BUSCO gene completeness. The main plot is divided into 1,000 size-ordered bins around the circumference with each bin representing 0.1% of the 691,289,278 bp assembly. The distribution of scaffold lengths is shown in dark grey with the plot radius scaled to the longest scaffold in the assembly (51,369,493 bp, shown in red). Orange and pale-orange arcs show the N50 and N90 scaffold lengths (25,204,545 and 16,195,774 bp), respectively. The pale grey spiral shows the cumulative scaffold count on a log scale with white scale lines showing successive orders of magnitude. The blue and pale-blue area around the outside of the plot shows the distribution of GC, AT and N percentages in the same bins as the inner plot. A summary of complete, fragmented, duplicated and missing BUSCO genes in the lepidoptera_odb10 set is shown in the top right. An interactive version of this figure is available at https://blobtoolkit.genomehubs.org/view/ilEudProf1.1/dataset/CAMQQK01/snail.

The final assembly has a total length of 691.3 Mb in 44 sequence scaffolds with a scaffold N50 of 25.2 Mb ( Table 1). Most (99.94%) of the assembly sequence was assigned to 28 chromosomal-level scaffolds, representing 27 autosomes and the Z sex chromosome. Chromosome-scale scaffolds confirmed by the Hi-C data are named in order of size ( Figure 2– Figure 5; Table 2). While not fully phased, the assembly deposited is of one haplotype. Contigs corresponding to the second haplotype have also been deposited. The mitochondrial genome was also assembled and can be found as a contig within the multifasta file of the genome submission.

**Table 1.** Genome data for *Eudemis profundana*, ilEudProf1.1.

Project accession data		
Assembly identifier		ilEudProf1.1	
Species		*Eudemis profundana*	
Specimen		ilEudProf1	
NCBI taxonomy ID		1100989	
BioProject		PRJEB56064	
BioSample ID		SAMEA10978938	
Isolate information		ilEudProf1, male, whole organism (genome sequencing and Hi-C scaffolding)	
Assembly metrics *		*Benchmark*
Consensus quality (QV)	65.8	*≥ 50*
*k*-mer completeness	100%	*≥ 95%*
BUSCO **	C:98.5%[S:97.4%,D:1.1%], F:0.4%,M:1.1%,n:5286	*C ≥ 95%*
Percentage of assembly mapped to chromosomes	99.94%	*≥ 95%*
Sex chromosomes	Z chromosome	*localised homologous pairs*
Organelles	Mitochondrial genome assembled	*complete single alleles*
Raw data accessions		
PacificBiosciences SEQUEL II		ERR10224931	
Hi-C Illumina		ERR10297825	
Genome assembly		
Assembly accession		GCA_947034925.1	
*Accession of alternate haplotype*		GCA_947034915.1	
Span (Mb)		691.3	
Number of contigs		135	
Contig N50 length (Mb)		16.6	
Number of scaffolds		44	
Scaffold N50 length (Mb)		25.2	
Longest scaffold (Mb)		51.4	

* Assembly metric benchmarks are adapted from column VGP-2020 of “Table 1: Proposed standards and metrics for defining genome assembly quality” from ( Rhie *et al.*, 2021). ** BUSCO scores based on the lepidoptera_odb10 BUSCO set using v5.3.2. C = complete [S = single copy, D = duplicated], F = fragmented, M = missing, n = number of orthologues in comparison. A full set of BUSCO scores is available at https://blobtoolkit.genomehubs.org/view/ilEudProf1.1/dataset/CAMQQK01/busco.

**Figure 3.**
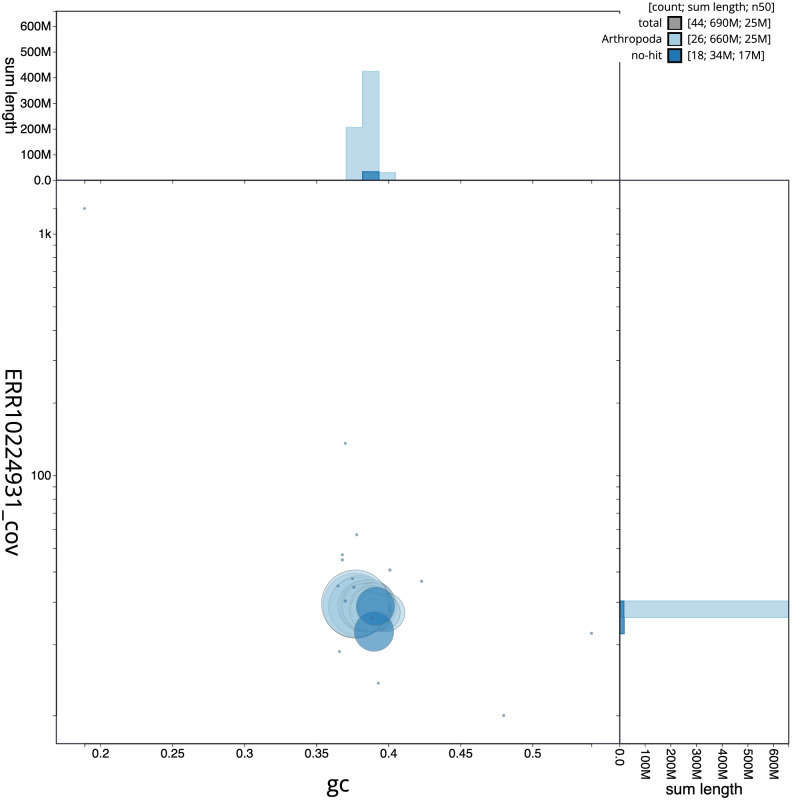
Genome assembly of *Eudemis profundana*, ilEudProf1.1: BlobToolKit GC-coverage plot. Scaffolds are coloured by phylum. Circles are sized in proportion to scaffold length. Histograms show the distribution of scaffold length sum along each axis. An interactive version of this figure is available at https://blobtoolkit.genomehubs.org/view/ilEudProf1.1/dataset/CAMQQK01/blob.

**Figure 4.**
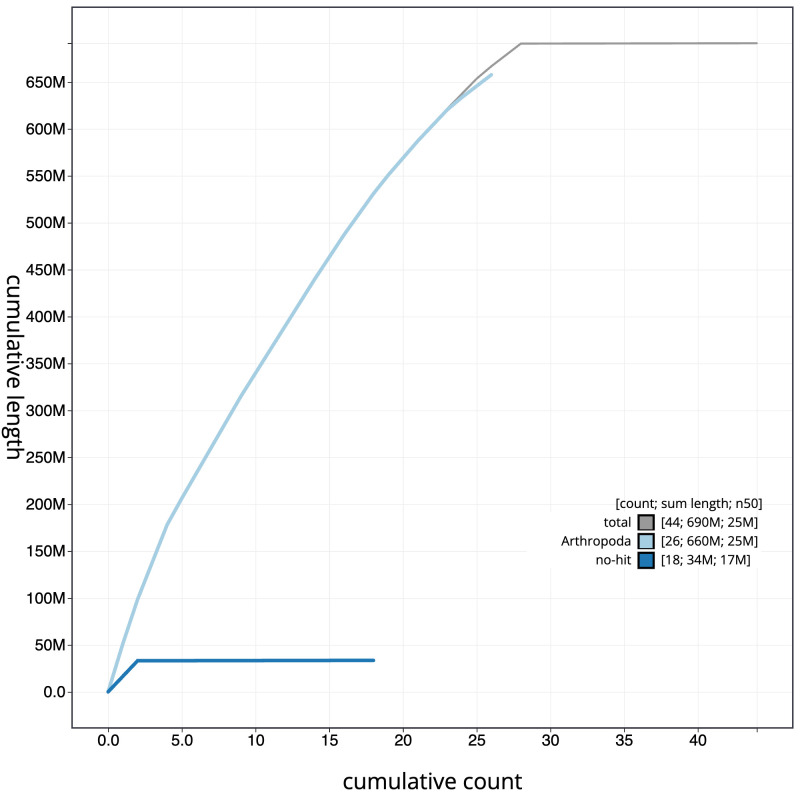
Genome assembly of *Eudemis profundana*, ilEudProf1.1: BlobToolKit cumulative sequence plot. The grey line shows cumulative length for all scaffolds. Coloured lines show cumulative lengths of scaffolds assigned to each phylum using the buscogenes taxrule. An interactive version of this figure is available at https://blobtoolkit.genomehubs.org/view/ilEudProf1.1/dataset/CAMQQK01/cumulative.

**Figure 5.**
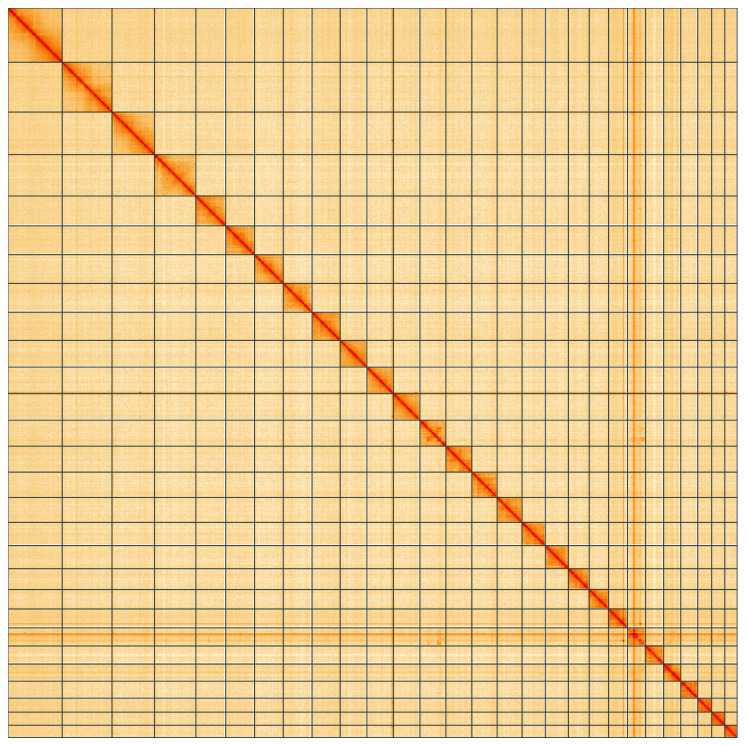
Genome assembly of *Eudemis profundana*, ilEudProf1.1: Hi-C contact map of the ilEudProf1.1 assembly, visualised using HiGlass. Chromosomes are shown in order of size from left to right and top to bottom. An interactive version of this figure may be viewed at https://genome-note-higlass.tol.sanger.ac.uk/l/?d=ZrVQpBgYT9utmKxSIJBcyQ.

**Table 2.** Chromosomal pseudomolecules in the genome assembly of *Eudemis profundana*, ilEudProf1.

INSDC accession	Chromosome	Size (Mb)	GC%
OX344795.1	1	47.19	37.7
OX344796.1	2	40.29	38
OX344797.1	3	39.16	37.9
OX344798.1	4	28.27	38.3
OX344799.1	5	27.3	38.7
OX344800.1	6	27.27	38.3
OX344801.1	7	27.2	38.2
OX344802.1	8	26.76	38.7
OX344803.1	9	25.23	38.6
OX344804.1	10	25.2	38.4
OX344805.1	11	25.02	38.7
OX344806.1	12	24.69	38.9
OX344807.1	13	24.57	38.7
OX344808.1	14	23.96	38.3
OX344809.1	15	23.59	38.4
OX344810.1	16	22.02	39
OX344811.1	17	21.88	38.8
OX344812.1	18	19.77	38.9
OX344813.1	19	18.37	38.8
OX344814.1	20	17.9	39.2
OX344815.1	21	17.14	39
OX344816.1	22	17.11	38.7
OX344817.1	23	16.2	39.8
OX344818.1	24	16.07	39.1
OX344819.1	25	13.26	39.6
OX344820.1	26	12.42	39
OX344821.1	27	11.7	39
OX344794.1	Z	51.37	37.7
OX344822.1	MT	0.02	19

The estimated Quality Value (QV) of the final assembly is 65.8 with *k*-mer completeness of 100%, and the assembly has a BUSCO v5.3.2 ( Manni *et al.*, 2021) completeness of 98.5% (single = 97.4%, duplicated = 1.1%), using the lepidoptera_odb10 reference set ( *n* = 5,286).

Metadata for specimens, spectral estimates, sequencing runs, contaminants and pre-curation assembly statistics can be found at https://links.tol.sanger.ac.uk/species/1100989.

## Methods

### Sample acquisition and nucleic acid extraction

A male *Eudemis profundana* (specimen number: Ox001669, ToLID: ilEudProf1) was collected from Wytham Woods, Oxfordshire (biological vice-county Berkshire), UK (latitude 51.77, longitude –1.31) on 17 July 2021. The specimen was taken from woodland habitat by Douglas Boyes (University of Oxford) using a light trap. The specimen was identified by the collector and snap-frozen on dry ice.

The ilEudProf1 sample was prepared at the Tree of Life laboratory, Wellcome Sanger Institute (WSI). The sample weighed and dissected on dry ice with tissue set aside for Hi-C sequencing. Whole organism tissue was disrupted using a Nippi Powermasher fitted with a BioMasher pestle. DNA was extracted at the WSI Scientific Operations core using the Qiagen MagAttract HMW DNA kit, according to the manufacturer’s instructions.

### Sequencing

Pacific Biosciences HiFi circular consensus DNA sequencing libraries were constructed according to the manufacturers’ instructions. DNA sequencing was performed by the Scientific Operations core on Pacific Biosciences SEQUEL II (HiFi) instrument. Hi-C data were also generated from tissue of ilEudProf1 that was set aside for this purpose, using the Arima v2 kit and sequenced on the Illumina NovaSeq 6000 instrument.

### Genome assembly, curation and evaluation

Assembly was carried out with Hifiasm ( Cheng *et al.*, 2021) and haplotypic duplication was identified and removed with purge_dups ( Guan *et al.*, 2020). The assembly was then scaffolded with Hi-C data ( Rao *et al.*, 2014) using YaHS ( Zhou *et al.*, 2023). The assembly was checked for contamination and corrected as described previously ( Howe *et al.*, 2021). Manual curation was performed using HiGlass ( Kerpedjiev *et al.*, 2018) and Pretext ( Harry, 2022). The mitochondrial genome was assembled using MitoHiFi ( Uliano-Silva *et al.*, 2022), which runs MitoFinder ( Allio *et al.*, 2020) or MITOS ( Bernt *et al.*, 2013) and uses these annotations to select the final mitochondrial contig and to ensure the general quality of the sequence. To evaluate the assembly, MerquryFK was used to estimate consensus quality (QV) scores and *k*-mer completeness ( Rhie *et al.*, 2020). The genome was analysed within the BlobToolKit environment ( Challis *et al.*, 2020) and BUSCO scores ( Manni *et al.*, 2021; Simão *et al.*, 2015) were calculated. Table 3 contains a list of software tool versions and sources.

**Table 3.** Software tools: versions and sources.

Software tool	Version	Source
BlobToolKit	4.0.7	https://github.com/blobtoolkit/blobtoolkit
BUSCO	5.3.2	https://gitlab.com/ezlab/busco
Hifiasm	0.16.1-r375	https://github.com/chhylp123/hifiasm
HiGlass	1.11.6	https://github.com/higlass/higlass
Merqury	MerquryFK	https://github.com/thegenemyers/MERQURY.FK
MitoHiFi	2	https://github.com/marcelauliano/MitoHiFi
PretextView	0.2	https://github.com/wtsi-hpag/PretextView
purge_dups	1.2.3	https://github.com/dfguan/purge_dups
YaHS	yahs-1.1.91eebc2	https://github.com/c-zhou/yahs

### Ethics and compliance issues

The materials that have contributed to this genome note have been supplied by a Darwin Tree of Life Partner. The submission of materials by a Darwin Tree of Life Partner is subject to the Darwin Tree of Life Project Sampling Code of Practice. By agreeing with and signing up to the Sampling Code of Practice, the Darwin Tree of Life Partner agrees they will meet the legal and ethical requirements and standards set out within this document in respect of all samples acquired for, and supplied to, the Darwin Tree of Life Project. All efforts are undertaken to minimise the suffering of animals used for sequencing. Each transfer of samples is further undertaken according to a Research Collaboration Agreement or Material Transfer Agreement entered into by the Darwin Tree of Life Partner, Genome Research Limited (operating as the Wellcome Sanger Institute), and in some circumstances other Darwin Tree of Life collaborators.

## Data Availability

European Nucleotide Archive: *Eudemis profundana* (diamond-back marble). Accession number PRJEB56064; https://identifiers.org/ena.embl/PRJEB56064. ( Wellcome Sanger Institute, 2022) The genome sequence is released openly for reuse. The *Eudemis profundana* genome sequencing initiative is part of the Darwin Tree of Life (DToL) project. All raw sequence data and the assembly have been deposited in INSDC databases. The genome will be annotated using available RNA-Seq data and presented through the Ensembl pipeline at the European Bioinformatics Institute. Raw data and assembly accession identifiers are reported in Table 1.

## Author information

Members of the University of Oxford and Wytham Woods Genome Acquisition Lab are listed here: https://doi.org/10.5281/zenodo.4789928.

Members of the Darwin Tree of Life Barcoding collective are listed here: https://doi.org/10.5281/zenodo.4893703.

Members of the Wellcome Sanger Institute Tree of Life programme are listed here: https://doi.org/10.5281/zenodo.4783585.

Members of Wellcome Sanger Institute Scientific Operations: DNA Pipelines collective are listed here: https://doi.org/10.5281/zenodo.4790455.

Members of the Tree of Life Core Informatics collective are listed here: https://doi.org/10.5281/zenodo.5013541.

Members of the Darwin Tree of Life Consortium are listed here: https://doi.org/10.5281/zenodo.4783558.
