# Supplementary material for: The genome sequence of the Diamond-back Marble, Eudemis profundana (Denis & Schiffermüller, 1775)
Source: Wellcome Open Res. 2023 Apr 25;8:184. [Version 1] doi: 10.12688/wellcomeopenres.19370.1 (PMC10904960; doi:10.12688/wellcomeopenres.19370.1)
Supplement: Peer review history

# Reviewer report: Reviewer response for version 1

**DOI:** 10.21956/wellcomeopenres.21457.r72292

**Published:** 2024-02-27

**Authors:** Balaji Chattopadhyay

**Affiliations:** 1 Ashoka University, Sonipat, Haryana, India

The manuscript presents a high-quality genome assembly of the Diamond-back Marble.

 The methods and results are fairly detailed and can serve as a methodological reference for many researchers who are implementing genome sequencing projects.

 I feel that the methods section can be improved slightly. The authors can add brief justifications for using specific programs. Additionally, they can provide parameters used for the analyses and brief clarification for using these specific parameters.

 The figure legends can include brief details about the results displayed.

Are sufficient details of methods and materials provided to allow replication by others?

Partly

Is the rationale for creating the dataset(s) clearly described?

Yes

Are the datasets clearly presented in a useable and accessible format?

Yes

Are the protocols appropriate and is the work technically sound?

Yes

Reviewer Expertise:

Genomics, NGS, Biodiversity, Evolution

I confirm that I have read this submission and believe that I have an appropriate level of expertise to confirm that it is of an acceptable scientific standard.

# Reviewer report: Reviewer response for version 1

**DOI:** 10.21956/wellcomeopenres.21457.r72291

**Published:** 2024-02-13

**Authors:** Merid Negash Getahun

**Affiliations:** 1 International Centre of Insect Physiology and Ecology, Nairobi, Kenya

In this short report, the authors present the genome from a single male of *Eudemis Profundana, *an Eurasian Lepidopteran specie. The article represents a good genome note although the findings have not been fully discussed. While the method and analyses are well done in general. We can take as a good resource if someone wants to know more about the species by analyzing the available data.

 Define the hypothesis why you are doing this work?

 Paragraph 2 of background need improvement it is confusing.

 The methodology does not clarify how the collected specimen was identified to the species level. This should be mentioned in the method. Also better photo of the insect with better describing features

 A bit more description of each figure to make it simple or understandable.

 Can we divide fig 3 in to 3 panels (A-C) and describe better each, what is the total we do not see it on the figure we can see it in the next figure.

 Figure 5 put the chromosome name and the scale bar showing how strong is the correlation. There is also an additional line that need to be removed or described. Except the identical comparison we cannot see any difference between other chromosomes. Add a legend describing the heatmap.

 Is it possible to conduct some phylogenomic analysis using selected gene and available data on the lepidopteran genome?

 It will be interesting to have some functional annotations (unless this was beyond the scope of the study).

 All The figures could be improved to make it readable for non-experts. Figure 5 needs a legend describing the heatmap.

Are sufficient details of methods and materials provided to allow replication by others?

Partly

Is the rationale for creating the dataset(s) clearly described?

Partly

Are the datasets clearly presented in a useable and accessible format?

No

Are the protocols appropriate and is the work technically sound?

Yes

Reviewer Expertise:

Insect Chemical ecology, Olfaction, Olafactory proteins, genes, vectors-host interaction

I confirm that I have read this submission and believe that I have an appropriate level of expertise to confirm that it is of an acceptable scientific standard, however I have significant reservations, as outlined above.

# Reviewer report: Reviewer response for version 1

**DOI:** 10.21956/wellcomeopenres.21457.r72290

**Published:** 2024-02-13

**Authors:** Jerome Hui

**Affiliations:** 1 Chinese University of Hong Kong, Hong Kong, Hong Kong

Boyes and colleagues report the genome sequence of a male diamond-back marble moth *Eudemis profundana* **(Denis and Schiffermuller, 1761). This species of moth can be commonly found in Britain. Molecular data of this species are scarce prior to this report, and are mainly confined to COI sequences deposited to the NCBI database. This new genome resource will be useful for further studies, such as identifying potential cryptic species, understanding its roles in the ecosystem, revealing its evolutionary relationships with other lepidopterans, and understanding their variable coloration in relations to the environment and perhaps also to climate change.

 This genome resource is excellent from the summary statistics, with high BUSCO numbers (98.5%), high sequence continuity (scaffold N50), and majority of sequences contained on the 28 pseudochromosomes (plus mitochondrion). To sum up, this is another valuable contribution.

Are sufficient details of methods and materials provided to allow replication by others?

Yes

Is the rationale for creating the dataset(s) clearly described?

Yes

Are the datasets clearly presented in a useable and accessible format?

Yes

Are the protocols appropriate and is the work technically sound?

Yes

Reviewer Expertise:

Genomics, evolution, invertebrates

I confirm that I have read this submission and believe that I have an appropriate level of expertise to confirm that it is of an acceptable scientific standard.
